# Perception of English Stress of Synthesized Words by Three Chinese Dialect Groups

**DOI:** 10.3389/fpsyg.2022.803008

**Published:** 2022-03-16

**Authors:** Xingrong Guo, Xiaoxiang Chen

**Affiliations:** ^1^College of Foreign Languages, Shanghai Maritime University, Shanghai, China; ^2^School of Foreign Languages, Hunan University, Changsha, China

**Keywords:** English stress, synthesized words, transfer, Chinese dialects, perception

## Abstract

This study investigated the possible prosodic transfer influences native regional dialects may have in the perception of English lexical stress by speakers of three Chinese dialects [Beijing (BJ), Changsha (CS), and Guangzhou (GZ)] compared to 20 American English (AE) speakers. F0, duration, intensity, and vowel reduction were manipulated in nonce disyllabic words. Participants performed four-word sequence recall tasks to identify lexical stress location. They performed better with natural sounds than with manipulated words. This study focused on the performance differences in manipulating words. The results showed that all four-group members performed similarly processing F0 condition nonce words. BJ and CS participants were more accurate than GZ participants in duration and vowel reduction cues. Reaction time (RT) suggested that the processing time of acoustic cues differed significantly across language groups. The findings indicate that first language (L1) dialect effect is robust in second language (L2) stress perception tasks.

## Introduction

English lexical stress plays a crucial role in native English speakers’ speech perception and production (Fry, 1955). Prior research suggests that second language (L2) learners of non-stress languages do not process stress as do native speakers (Zhang et al., 2008; Zhang and Francis, 2010). L2 learners may employ the stress placement strategy in L2 acquisition according to first language (L1) strategies (Wang, 2008).

Evidence indicated that stress perception is the result of the interaction of F0, duration, intensity, and vowel reduction (Chrabaszcz et al., 2014). Errors in these correlates may interfere with stress contrast production and perception (Fry, 1955). An L1 Chinese speaker often has difficulty producing or perceiving, native-like English lexical stress due to L1 prosodic transfer effects (Zhang et al., 2008; Zhang and Francis, 2010; Qin et al., 2017). Lexical tone sensitivity contributes to English stress sensitivity development (Choi et al., 2017).

In the past few decades, scholars have conducted considerable research on Chinese students’ English stress acquisition. Most of them focused on investigating the performance of L2 learners. However, cross-dialectal variation of L2 learners has been neglected. Is there any difference in acoustic correlates among speakers of different Chinese dialects in perception? Does acoustic cues of L1 dialect transfer to L2 perception? This study explores whether, or to what degree, L1 interferes with L2 participants’ English stress perceptions.

### Research Questions

This study intended to address these two questions:

Q1. Which acoustic cues: F0; duration; intensity; or, vowel reduction, do Chinese participants and English participants rely upon to identify stress when acoustic correlates were manipulated?
Q2. Whether L1 dialect background would influence L2 participants’ perception of lexical stress?

### Comparison of the Rhythm Typology Between English and Mandarin

Typologically, Chinese and English belong to distinctly different prosodic and rhythmic types. Most English words are polysyllabic (Mousikou et al., 2017). Mandarin is a monosyllabic language with a basic consonant-vowel syllable structure. Mandarin is a syllable-timed language. English utilizes all four acoustic correlates to cue word stress: segmental (vowel reduction) and suprasegmental (F0, duration, and intensity) cues. Mandarin tones differ mainly in F0 height and contour (Chen et al., 2001). Mandarin has full-full and full-reduced words that differ in stress placement. Segmental and suprasegmental cues signal stress (Connell et al., 2016). English is a stress-timed language. Inter-stress duration intervals are more consistent (Ramus et al., 1999). Given the rhythmic differences between these languages, English lexical stress acquisition by Mandarin speakers may involve learning to reduce, or shorten, unstressed syllables.

Whether Mandarin is a lexical stress language is still a matter of controversy. Duanmu (2004) argued that Mandarin also has a stress pattern that contrasts full-full and full-reduced disyllabic words. Duanmu (2007) argues that Mandarin full syllables, which are syllables having four lexical tones, are equivalent to English stressed syllables and that neutral tones are equivalent to English unstressed syllables. Neutral tones, often occurring in two, or more syllables, are less prominent than syllables having the four lexical tones. Full syllables sound louder and have a greater intensity and longer duration than neutral syllables. English has some minimal pairs such as ‘CONtract-con’TRACT. Similar stress pairs are in Mandarin. The minimal pair “dōng xī-dōng xi” is the same Chinese characters “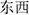,” but has two distinct meanings: (1) “East-West” and (2) “something.” The only difference is that both are stressed when meaning “East-West,” while the second syllable unstressed when meaning “something.” All Chinese dialects carry tones. Some are distinguished by whether they are, or are not, lexically stressed. Guangzhou (GZ) dialect does not have this stress distinction. GZ words have full-full patterns. The second syllable of disyllabic words is not reduced.

### Comparison of Beijing, Changsha, and GZ Dialects

Different Chinese dialects are not always mutually intelligible, even when situated within the same province (Yuan, 1989). Li (2015) found that Chinese dialect timing and melody patterns are distinct across dialects, and their prosodies are heterogeneous. This study chose participants from Beijing (BJ), Changsha (CS), and GZ dialects to investigate whether L1 dialect would influence the learners’ perception of English stress. Supplementary Table 1 presents a summary of the difference of tones in BJ, CS, and GZ dialects.

The Beijing dialect is the phonological basis for Mandarin, which is the official language of the People’s Republic of China and is a typical representative of northern dialects. There are four basic tones in Mandarin (T1, high flat; T2, high rising; T3, low dipping; and T4, falling). T1 and T2 are higher in BJ dialect, T3 dips more prominently, and T4 falls more. The neutral tone (light and short syllable) occurs very frequently in the BJ dialect. The F0 contours of the neutral tone are much less consistent than the full-tone syllables. F0 contour of the neutral tone changes with the tone of the preceding syllable (Lee and Zee, 2008). F0 is the most reliable acoustic cue for neutral tone perception (Fan et al., 2015). The duration of the neutral tone syllable was about half of the full-tone syllable (Lin and Yan, 1980). The salience hierarchy of the acoustic correlates of the BJ dialect tone is F0 > duration > intensity (Fu et al., 1998; Liu and Samuel, 2004).

The Changsha dialect is a new Xiang dialect and is heavily influenced by Southwestern Mandarin. CS dialect tones share features with Mandarin tones, with a slight difference. There are six tones mid (33), rising (13), falling (41), high (55), low (11), and checked (24). Yi (2007) found that, unlike the BJ dialect tone, duration plays a more important role in the CS dialect metrical stress than F0 and intensity. When distinguishing CS dialect lexical stress, intensity and vowel reduction do not play a role (Yi, 2007). The CS dialect lexical stress acoustic correlate salience hierarchy is duration > F0 > intensity (Yi, 2007). “The duration of the neutral-toned syllable is about 60–70% as that of the full-tone syllable” (Zhang, 2005).

Guangzhou is a tonally rich dialect having no neutral tone. GZ dialect is well-known for its rich tones and similarity among tone contours: high-level (55), mid-rising (35), mid-level (33), low-falling (21), low-rising (13), low-level (22), high-level (5), mid-level (3), and low-level. The last three tones have the same pitch as 55, 33, and 22, respectively. Recent studies on GZ dialect phonology contend that GZ dialect has only six tones rather than nine tones. These studies considered Tone (T) 7, T8, and T9 as carrying the same tone level as T1, T3, and T6, respectively (Matthews and Yip, 2013). Each GZ dialect syllable has a lexical tone and receives almost equal emphasis (Bauer and Benedict, 1997). F0 is the primary acoustic cue in Cantonese tones (Tong et al., 2015). GZ dialect and the BJ dialect tone “differ dramatically in precise F0 range, length, and endpoints” (Yeung et al., 2013). F0 is “the primary, and perhaps sole, cue to lexical tones in Cantonese” (Ciocca et al., 2002).

Neutral tones occur in BJ and CS dialects, but not in GZ. BJ and CS dialect neutral tones occur at the final position of a word and are produced in a light and short way. The GZ dialect syllable-timing was reported to be much stronger than the BJ dialect since the GZ dialect has a simple syllable structure without lexical stress and phonological vowel reduction (Mok, 2009).

### Prior Studies of English Stress Perception

Non-native speech sound perception is greatly influenced by native L1 prosodic knowledge (Chrabaszcz et al., 2014). This perceptual bias has been repeatedly observed in L2 acquisition. Japanese listeners have difficulties in discriminating between the English/r/–/l/contrasts. They perceive these contrasts as phoneme variants of their L1 (Miyawaki et al., 1975). L1–L2 interference occurs in segmental contrasts and in their suprasegmental dimensions. L1 influences lexical stress perception through stress patterns and acoustical cues.

Previous studies showed that speakers whose language has contrastive stress have relatively little difficulty in processing L2 stress. Cooper et al. (2002) used four cross-modal priming experiments and two forced-choice identification experiments to investigate Dutch-speaking learners of English. They utilized stress to distinguish Dutch words and successfully used English stress suprasegmental cues to distinguish English words. Dupoux et al. (2008) found that Spanish L1 learners of English successfully recognized and recalled the nonce words that differ in stress placement. Peperkamp and Dupoux (2002) found that French-speaking English learners, who usually stressed the terminal word ultimate syllable in a phrase had difficulty distinguishing nonce word stress. Those researchers attributed this to L2 learners’ inability to recognize stress contrast as determined by L1 stress parameters (Peperkamp and Dupoux, 2002).

Numerous studies have found that speakers of non-stress languages have difficulties perceiving lexical stress (Lin et al., 2013). Prior studies on Mandarin L1 speakers’ perception of English lexical stress have yielded a variety of findings. Zhang and Francis (2010) used a forced-choice stress pattern identification task to examine the weight of Mandarin L1 learners and native speakers of English use to process English lexical stress. Mandarin speakers with a mid-to-high English proficiency level relied more on vowel reduction than on suprasegmental cues (i.e., F0, duration, and intensity) to English stress and did not differ from American listeners in utilizing F0 and duration cues. Wang (2008) conducted a forced-choice English stress identification study. Wang reported that Mandarin-speaking learners of English did not rely on segmental cues. They mainly relied on F0 to identify English stress. These inconsistent findings were probably due to L2 learners’ different proficiencies. Prior research indicates that L2 speakers adeptly use acoustic cues present in the target language if these correlates are actively applied for realizing L1 prosodic contrasts. Alan (1990) found that CS participants produced English stress as tone and they used tone one (T1) with an inordinate degree of length to indicate stressed syllables. Qin et al. (2017) report that Standard-Mandarin (SM) and Taiwan-Mandarin (TM) speakers performed similarly in using F0 to perceive English stress. SM used duration more than TM speakers. SM uses duration together with F0 to realize lexical stress. TM has no stress distinctions. They attributed it to that TM has neutral tone, which instantiates lexical stress by duration. TM does not have this distinction. They concluded that L1 dialect plays an important role in determining whether non-native listeners could use specific acoustic cues to encode English stress. Guo and Chen (2017) discovered that BJ dialect participants’ stress perception resembled that of the English participants than did GZ participants. These findings suggest that L1 dialects may transfer to L2 English stress perception.

### Cue-Weighting Theory

Cue-weighting is “a useful methodological tool in speech perception research: it allows to access within-group and between-group biases in sound categorization” (Kapatsinski et al., 2011). The cue-weighting theory (CWT) (Francis and Nusbaum, 2002; Holt and Lotto, 2004, 2006; Zhang and Francis, 2010; Qin et al., 2017) accounts for this speech perception and calls for attention to its phonetic features. It focuses on how the weighting of acoustic cues stress contrasts in foreign languages and how these weighting variations influence L2 speech perception and processing (Qin et al., 2017).

The CWT predicts that speech perception is multidimensional and that acoustic cues are weighted dissimilarly across different languages or different categories. Though multiple cues are simultaneously available to listeners, they are weighted differently and often show a “trading relation” (Repp, 1982). When one cue sensitivity increased, another cue sensitivity often decreased. L2 learners’ attendance to dissimilar acoustic cues when perceiving the same stimuli hinges on how these acoustic cues are utilized to signal contrast in their L1.

## Methodology

Following previous studies on the processing of stress (Dupoux et al., 2001, 2008; Qin and Tremblay, 2014; Qin et al., 2017), this study’s paradigm is based on a short-term sequence recall experiments involving stress processing. Nonce words were used here to avoid interference from real words the participants might have memorized.

### Participants

A total of 20 native American English (AE) speakers were included as subjects in this study. Their age range was 21–27, *M* = 22.7. They had no tonal language background. A total of 60 Chinese speakers also participated. The Chinese participants were university students from the People’s Republic of China. The Chinese participants were divided into three groups. The age range was 16–23, *M* = 18.9. They were divided into BJ, CS, and GZ groups. None had ever resided in an English-speaking country. There were 10 men and 10 women in each group. No participant had any diagnosis of a cognitive or speech disorder. They all filled out written informed consent before participating in the experiment.

Prior to enrollment, participants completed language background questionnaires. Each Chinese participant completed a longer questionnaire. It included information about English learning experience, age at L2 acquisition, and language usage information. Each AE participant completed a shortened questionnaire. Chinese participants rated their English proficiency level, on a 10-point Likert scale, for several domains, including pronunciation, vocabulary, and grammar. These are presented in Supplementary Table 2.

The L1 influence on L2 acquisition is constrained in many ways. Chinese dialect groups had roughly intermediate English proficiency levels. Chen and Guo (2017) reported that L2 learners produced less native-like stress patterns. Those acoustic values varied according to L2 proficiency. All Chinese L1 learners of English used F0, duration, and intensity to identify stress. L2 learners varied in their use of duration, possibly due to L1 tonal transfer. Speaker demographics and proficiency information are presented in Supplementary Table 3.

**FIGURE 1:**
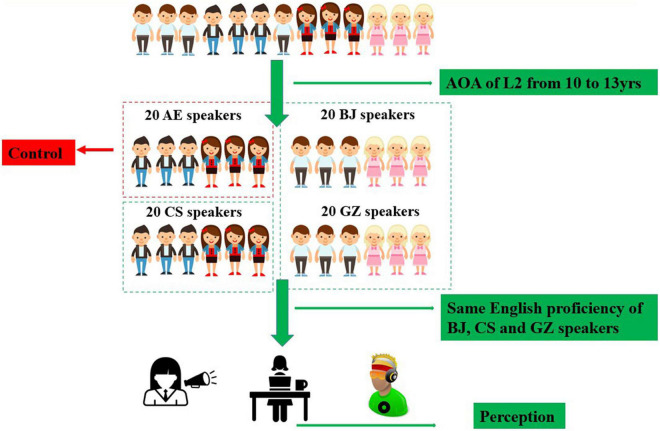
The procedure for selecting participants.

All Chinese dialect-speaking participants grew up in monolingual homes and had not begun learning English prior to school education. Chinese participants mainly received English language instruction in school. This averaged a duration of 13.1 years for BJ participants, 10.4 years for CS participants, and 10.2 years for GZ. They only spoke English in their English classes. Figure 1 shows the procedure of selecting participants.

Selection criteria included age at L2 acquisition, listening and proficiency levels, and daily life L1 and L2 use. Chinese participants’ English score of college entrance examination scores ranged from 110 to 115 out of a maximum of 150. Chinese participants’ English proficiency was measured using the *Language Experience and Proficiency Questionnaire* (Marian et al., 2007) and the *Lexical Test for Advanced Learners of English (LexTALE)* (Lemhfer and Broersma, 2012). An advantage of using written rather than aural, or oral, proficiency tests is that they avoid potentially circular argumentation by tapping into some language skills that are highly predictive of general proficiency. This is different from phonological processing task skills currently being used. Performance in an aural, or oral, comprehension experiment seems to be affected by listener abilities to perceive and produce English stress. The *LexTALE* is accepted as a valid and reliable measure of English proficiency. Intermediate English proficiency levels were invited to participate. One-way ANOVA results suggest that selected Chinese participants did not differ in proficiency ratings or lexical test scores (all *p*s > 0.05).

### Stimuli

Minimal pair English nonce words having differing stress, such as/’fΛði/and/fΛ’ði/, were the stimuli. They were all disyllabic words having the same consonant-vowel structure (C_1_V_1_ C_2_V_2_)./I/,/ʊ/, and/Λ/were used in the V_1_ position./i/was used in V_2_ position. These sounds do not reduce to schwa, which assured that the target stimuli would not have vowel reduction and still sound like real English words. Four fricatives (/θ/,/v/,/f/and/ð/) were used for C_1_ and C_2,_ so that consonants would not provide segmental cues for stress (Qin et al., 2017).

Using the stimuli in Qin et al. (2017), four segmental non-words:/sIvi/,/zʊθi/,/fΛði/, and/hΛfi/, which were used for analyzing the influence of F0, duration, and intensity. Four non-words:/sava/,/zaθa/,/fasa/, and/hafa/, in which non-stressed vowels reduce to shwa, were recorded to explore the use of the vowel reduction cue for the perception of lexical stress (Wang, 2016). Adapting the stimuli used in Wang (2016) and Qin et al. (2017), about 16 experimental nonce words were used as target stimuli (Supplementary Table 4).

Adapting the stimuli used in Qin et al. (2017), this study included fillers to avoid specific stress processing strategies. Filler stimuli had different initial word consonants. They always contrasted/t/and/k/ [e.g., (‘tΛfi) and (kΛ’fi)] in the C_1_ position and used/I/,/ʊ/, and/Λ/in V_1_ position and/i/in the V_2_ position (Qin et al., 2017). These two consonants were used to prevent learners from developing processing strategies for stress judgments. The filler stimuli were contrasted by/t/and/k/. Voiced stops (/b/,/d/, and/g/), sonorants (/m/), or fricatives (/f/,/v/,/θ/, and/ð/), were chosen for the C_2_ position to enrich the phonetic variability in the filler stimuli. Sixteen filler stimuli are listed in Supplementary Table 5 (adapted from Wang, 2016 and Qin et al., 2017). The filler stimuli encouraged the listeners to use both suprasegmental and segmental information (Qin et al., 2017). All stimuli were produced by an AE male speaker. Each non-word was recorded four times in the carrier sentence: “Please say X CLEARLY but not LOUDLY.” Nonce word acoustic measurements are presented in Table 1, indicating that the nonce words with an initial and a final stress pattern differed significantly in the V_1_/V_2_ ratios of F0, duration, and intensity.

**TABLE 1:** Mean values of nonce words.

Cue	Value	Trochee			Iamb		
			
		V_1_	V_2_	Ratio	V_1_	V_2_	Ratio
F0	Mean	201	116	1.7	132	175	0.8
	SD	16	11		6	18	
Duration	Mean	183	140	1.3	104	142	0.7
	SD	12	23		49	30	
Intensity	Mean	73	57	1.3	51	58	0.9
	SD	2	2		10	9	

Nonce words having initial stress differed significantly for the F0 cue (*p* < 0.01) (Table 1). For trochee, the V_1_/V_2_ ratio is 1.7, whereas, in the iamb, the V_1_/V_2_ ratio is 0.8. Nonce words with initial stress and final stress differed significantly in duration cue (*p* < 0.01). In trochee, the V_1_/V_2_ ratio is 1.3, whereas, in iamb, the V_1_/V_2_ ratio is 0.7. Nonce words with initial stress and final stress differed significantly in intensity cue (*p* < 0.01). In trochees, the V_1_/V_2_ ratio is 1.3, whereas, in iambs, the V_1_/V_2_ ratio is 0.9. Table 2 presents the vowel quality of nonce words in trochaic and iambic stress patterns.

**TABLE 2:** Nonce word vowel quality.

Value	Trochee				Iamb			
		
	V_1_		V_2_		V_1_		V_2_	
				
	F1	F2	F1	F2	F1	F2	F1	F2
Mean	554	1,517	281	2,216	485	1,542	312	2,276
SD	74	42	36	110	67	73	14	33

Nonce words with initial stress and final stress differed significantly in vowel reduction cue (*p* < 0.01) (Table 2). F1 and F2 values in trochee were significantly different from iamb.

### Manipulation

This section describes the systematic manipulation of the four acoustic cues: F0, duration, intensity, and vowel reduction. Normalization and manipulation scripts were adapted according to Wang (2008). All recorded items were digitized at 44.1 kHz, 16 bits, digitally edited. According to the previous study on English stress by Fry (1955, 1958), the variation of F0, duration, intensity, and vowel reduction to signal stress is mainly on the vocalic portion of a syllable. The manipulations of the acoustic cues were applied to the vowel nuclei of the disyllabic words (V_1_ and V_2_).

First, nonce words were segmented and annotated using Praat scripts. Then, the second procedure is normalization. Three Praat scripts were used for normalization in the following order: (1) duration normalization script; (2) F0 normalization script; and (3) intensity normalization script.

Duration normalization script was used to measure the duration of V_1_ and V_2_. The two vowels were then normalized to have the same duration. F0 normalization script was used to normalize the V_1_ and V_2_ pitch contour. First, a sound was segmented into five intervals. Then, the pitch contour of five intervals was obtained by using the pitch average script. Then, a pitch value was obtained every 0.01 s to the end of the vowel.

Similar to the manipulation process of F0, an intensity normalization script was run to generate the same intensity contour of V_1_ and V_2_. First, the highest intensity of the two vowels was gotten by using the intensity average script. Then, the average intensity value was calculated. All these stimuli were normalized to get the same F0 contour, duration, and intensity contour. These normalized stimuli were used for further manipulation.

The experimental stimuli were resynthesized based on the average values (Tables 1, 2). Under the condition of “all cues,” these four cues (F0 + duration + intensity + vowel reduction) were all used to signal English stress. Under the condition of “F0 cue,” F0 was the only hint of stress position. The duration and intensity of V_1_ and V_2_ were normalized. Under the condition of “duration cue,” the duration signaled stress. F0 and intensity were normalized. Under the condition of “intensity cue,” intensity was used to signal stress. F0 and duration of the two syllables were normalized. Under the condition of “vowel reduction cue,” only vowel reduction signaled stress. Except for the vowel reduction, the other three acoustic correlates were normalized. Manipulation was first implemented on the first vowel and then on the second vowel. All cues were manipulated using Praat scripts (Boersma and Weenink, 2014).

### Procedure

Participants were tested individually in sound-attenuated booths. The presentation order was randomized for each listener by E-prime 2.0. Sequence recall tasks had two phases: familiarization and testing. The three buttons labeled “F,” “J,” and “space bar” on the keyboard were the only functioning keys during the experiment. Participants should press “F” if they think the first syllable is stressed, “J” if the second syllable is stressed. The space bar is pressed to continue. Participants had 5 min to learn how to associate with the keyboard “F” and “J” with initial and final stress. Participants were provided with “Correct” and “Incorrect” feedback. Real words with minimal stress pairs were used in the familiarization phase. Once a participant obtained a 95% consecutive correct rate, they proceeded to the testing phase. If a participant was unable to obtain 95% accuracy, they were retrained. Initially, 74 of 80 participants obtained the required accuracy rate. Unsuccessful participants underwent additional training. The familiarization task took 5–15 min depending on whether the participants achieved the accuracy criterion level during the initial attempt.

Each trial began with the fixation sign “+” being displayed for 500 ms to the participant. A four-word sequence was heard. There was an interstimulus interval of 50 ms (Dupoux et al., 2001, 2008). The words “OK” then followed to avoid participants from echoic memory interference. The inter-trial interval for responses was 5,000 ms. Participants pressed four keys after hearing a four-word sequence. Success was defined as four correct responses in a row. Would-be participants were allowed to participate in the testing phase only after they proved their competence. The four-item sequences were adopted to prevent floor and ceiling effects. Each four-word sequence included two stimuli with a trochaic stress pattern and two stimuli with an iambic stress pattern. There were six possible token orders [i.e., (1,122), (2,211), (1,212), (1,221), (2,121), (2,112)]. The experiment included a total of 480 experimental trials = (16 nonce words × 6 orders × 5 manipulations).

### Data Analysis

Participant responses were classified as follows: For data analyses, (1) only responses that were 100%—correct transcriptions of the four-word sequences were coded as correct; (2) all other responses were coded as incorrect. Responses that were 100%—incorrect transcriptions were coded as reversals. Participants’ results with more reversals than correct responses were excluded from consideration. According to the previous research (Dupoux et al., 2001, 2008; Qin et al., 2017), the high percentages of incorrect responses were due to the potential transposition of directions. For example, some participants mistakenly associated the “F” on the keyboard with the unstressed syllable. This criterion resulted in the exclusion of one CS and two BJ participants from the phoneme contrast and three AE participants from the comparison.

*Logit mixed-effects* models were used to analyze the participant sequence-encoding accuracy. All subsequent analyses were conducted in *R* studio (Gentleman and Ihaka, 2011), using the *lme 4* package for mixed-effects models.

The section “Results” will first show the participants’ accuracy and then the reaction time (RT). Two models were used to analyze participants’ accuracy. The first model analyzed the sequence-encoding accuracy of the four language groups, with L1 (AE, BJ, CS, and GZ; control group: AE) as fixed effect and with participant, test item, trial order, and sequence order as crossed random effects. The second model analyzed the accuracy of L2 participants. Estimates represent accuracy change *log* odds caused by changes in the language group, condition variables, and values of *p*, which are based on the *Wald z* distribution.

## Results

### Accuracy Rates

**FIGURE 2:**
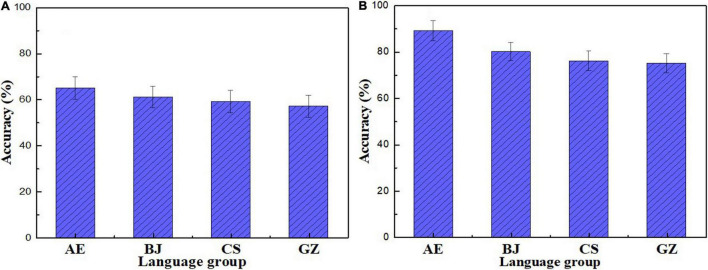
Stimuli **(A)** and filler **(B)** sequence-encoding accuracy.

The sequence-encoding accuracy of the four language groups is shown in Figure 2.

Figure 2A shows that all participants performed above the chance level. The order is AE > BJ > CS > GZ. The *logit mixed-effects model* for Chinese L1 participant’ accuracy did not include any fixed effect such as adding L1 to the model. This suggests that the three Chinese L1 groups did not differ from the AE group in encoding English lexical stress.

**FIGURE 3:**
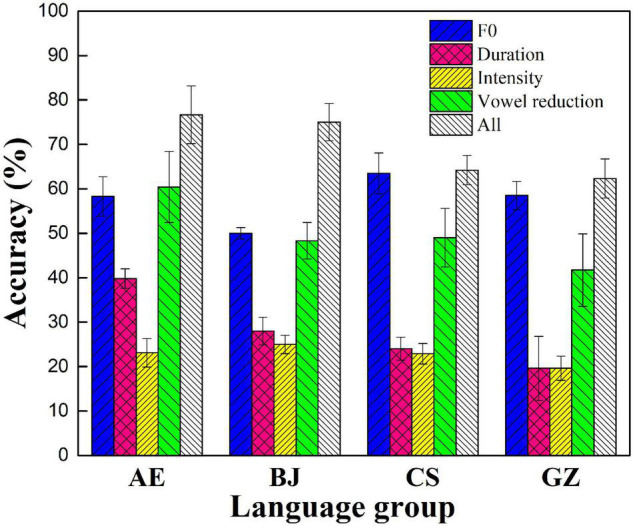
Participants’ sequence-encoding accuracy of the five conditions.

All groups performed above the chance level (Figure 2B). Accuracy rank order is AE > BJ > CS > GZ. The best logit mixed-effects model for accuracy included L1 as a fixed effect (Supplementary Table 6). Filler stimuli results were that the AE group outperformed the BJ, CS, and GZ groups in perceiving/t/and/k/in English nonce words. The best logit mixed-effects model on the L2 participants’ accuracy did not include any effect, suggesting that BJ, CS, and GZ participants did not differ from each other in their encoding of/t/and/k/in English nonce words. Accuracy in the sequence recall task in the five conditions is shown in Figure 3.

As shown in Figure 3, the four language groups all performed above the chance level in the five conditions. AE, BJ, and GZ groups all performed the best in all-cue conditions among the five conditions. For the AE group, the ranking order of the accuracy in the five conditions is as follows: all cues > vowel reduction > F0 > duration > intensity; BJ: all cues > F0 > vowel reduction > duration > intensity; CS: F0 > all cues > vowel reduction > duration > intensity; and GZ: all cues > F0 > vowel reduction > duration > intensity.

Supplementary Table 7 presents the results of the accuracy of the sequence recall task of the perception experiment. The *logit mixed-effects models* on all the participants’ accuracy in the five conditions included L1 language, cue condition, and their interaction as fixed effects. Fixed effects include cue conditions (F0 cue, duration cue, intensity cue, vowel reduction cue, baseline = F0 + duration + intensity + vowel reduction cues); L1 (AE, BJ, CS, GZ; baseline = AE). The interaction between the two is considered as fixed effects. Random effect includes participants, test item, trial order, and sequence order. The results of this model are summarized in Supplementary Table 8. The model showed significant influences of F0 [*z* (2,520) = 6.73, *p* < 0.001], duration [*z* (2,520) = 4.13, *p* < 0.0001], and vowel reduction [*z* (2,520) = 4.88, *p* < 0.001] cue conditions, but not intensity cue [*z* (2,520) = 1.38, *p* = 0.17]. There were significant interactions between duration cue and CS dialect, *z* (2,520) = − 2.39, *p* = 0.02, as well as significant interactions between all-cues condition and CS dialect, *z* (2,520) = − 2.27, *p* = 0.02. There was no significant difference among the other groups and other acoustic cues.

The *logit mixed-effects models* on Chinese dialect participants’ accuracy in the five prosodic conditions included L1 language, cue condition, and their interaction as fixed effects. Supplementary Table 9 presents a summary of the results of this model. The results showed a significant effect of F0 cue [*z* (2,520) = 6.70, *p* < 0.0001], duration cue [*z* (2,520) = 4.15, *p* < 0.001], vowel reduction cue [*z* (2,520) = 4.82, *p* < 0.0001], but not intensity cue [*z* (2,520) = 1.44, *p* = 0.15]. There were significant interactions between all-cue condition and BJ dialect [*z* (2,520) = − 2.23, *p* = 0.03], duration, and CS dialect [*z* (2,520) = − 2.35, *p* = 0.02], as well as significant interactions between all-cue condition and CS dialect, *z* (2,520) = − 2.23, *p* = 0.03. However, other significant differences were not found. These results indicate that in the presence of different cue conditions, Chinese participants showed a distinct preference for the relative reliance on different acoustic cues.

**FIGURE 4:**
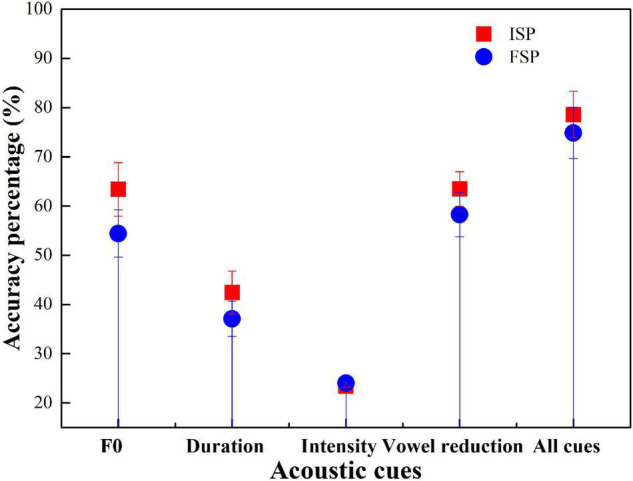
Accuracy for Initial Stress Percentage (ISP) and Final Stress Percentage (FSP) with the American English (AE) group.

Participant performance similarities in F0, duration, intensity, and vowel reduction manipulations, the Initial Stress Percentage (ISP), and the Final Stress Percentage (FSP) were calculated. Four univariate ANOVAs were conducted for the four groups. The dependent variable was ISP, with the fixed factors being F0, duration, intensity, and vowel reduction. Figure 4 shows the accuracy of ISP and FSP using the four groups.

For most cases, except the intensity cue condition, the accuracy of ISP was higher than FSP (Figure 4). ANOVA results showed that F0 had a significant effect on AE stress judgment (*p* < 0.001). Duration had a significant effect (*p* < 0.001). Intensity showed no significant effects (*p* = 0.87). Vowel reduction had a significant effect (*p* < 0.001). All-cue condition showed a significant effect (*p* < 0.001).

**FIGURE 5:**
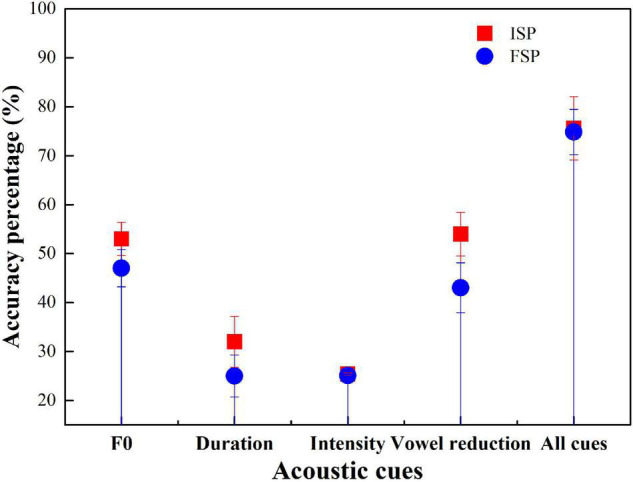
Accuracy for ISP and FSP with the BJ group.

Initial Stress Percentage accuracy was greater than FSP in F0, duration, vowel reduction, and all-cue condition, but not in intensity condition (Figure 5). ANOVA results showed that F0 had no significant effect on BJ stress judgment (*p* > 0.05). Duration had a significant effect (*p* < 0.05). Intensity showed no significant effect (*p* = 0.89). Vowel reduction had a significant effect (*p* < 0.05). The all-cue condition showed no significant effect (*p* = 0.67).

**FIGURE 6:**
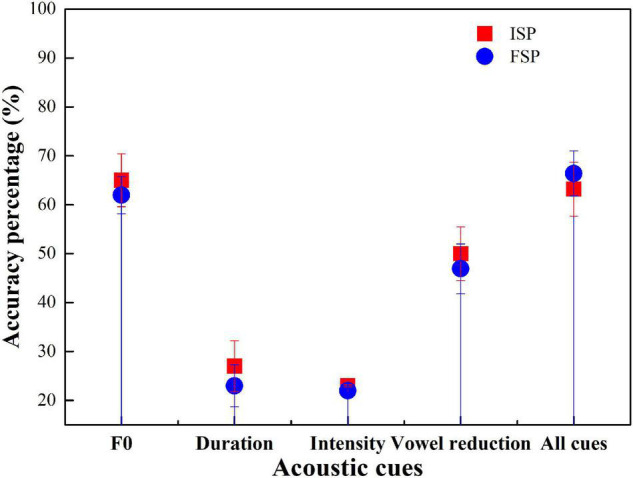
Accuracy for ISP and FSP with the CS group.

Initial Stress Percentage accuracy was greater than FSP in F0, duration, intensity, and vowel reduction, but not all-cue conditions (Figure 6). ANOVA results showed that F0, duration, intensity, vowel reduction, and all-cue conditions showed no significant effect on CS stress judgment (*p* > 0.05). Duration showed a significant effect (*p* < 0.05).

**FIGURE 7:**
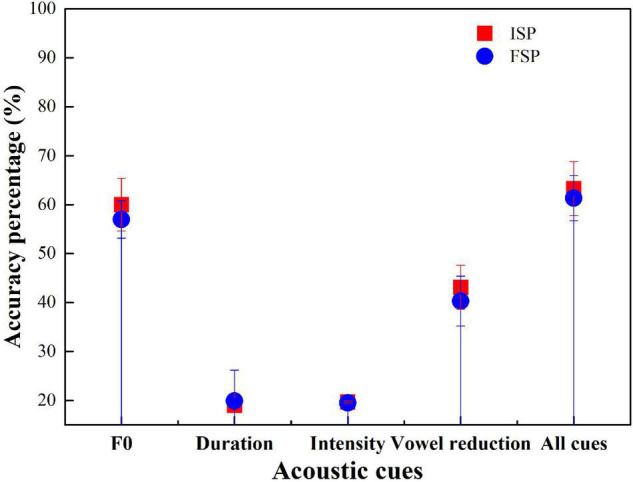
Accuracy for ISP and FSP with the GZ group.

Initial Stress Percentage accuracy was greater than FSP in F0, vowel reduction, and all-cue condition, but not in duration and intensity conditions (Figure 7). ANOVA results showed that F0, duration, intensity, vowel reduction, and all-cue conditions showed no significant effect on GZ stress judgment (*p* > 0.05).

### Reaction Times

**FIGURE 8:**
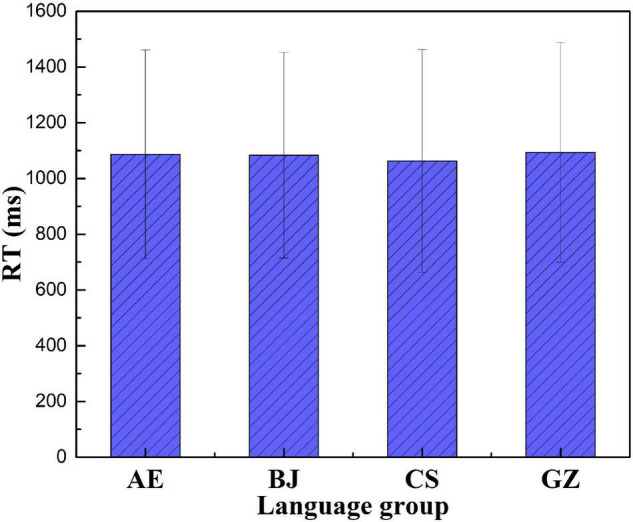
Average participant stimuli reaction time (RT).

It has been argued that RT shed light on how a participant processes language. RT was frequently used as a measure to indirectly reflect processing stress. The assumption is that the longer it takes for a participant to respond to a stimulus, the more processing “energy” is required. RT data for incorrect responses were not included in the data analysis. RT data < 300 ms and RT > 2,000 ms were excluded (Jiang, 2013). In all, 18.6% of the data (2.3% AE, 7.6% BJ, 2.4% CS, and 6.3% GZ) were excluded. All RT data were log-transformed to improve normality. Figure 8 shows the means of accuracy and RT for nonce words across the four language groups.

**FIGURE 9:**
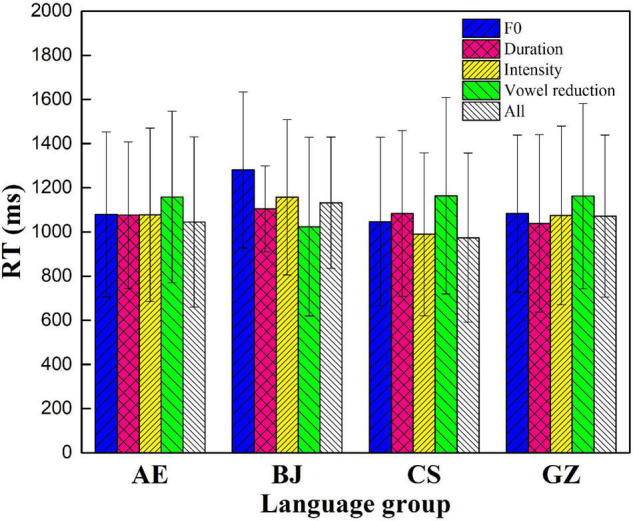
Participant RT for the five conditions.

Guangzhou participants performed the slowest in the four language groups (Figure 8). The ranking order of the RT was as follows: GZ (1,094) > AE (1,087) > BJ (1,083) > CS (1,063). Figure 9 shows the participants’ RT in the five conditions.

The RT ranking order in the five conditions was as follows (Figure 9). AE: vowel reduction (1,158) > F0 (1,079) > intensity (1,078) > duration (1,076) > all conditions (1,045); BJ: F0 (1,281) > intensity (1,157) > all conditions (1,132) > duration (1,105) > vowel reduction (1,023); CS: vowel quality (1,164) > duration (1,084) > F0 (1,047) > intensity (990) > all cues (974); and GZ: vowel reduction (1,162) > F0 (1,084) > intensity (1,075) > all cues (1,071) > duration (1,039).

Supplementary Table 10 presents a summary of the average RT in the five conditions. Different groups had English lexical stress processing speeds, suggesting that they used different strategies. With process F0 cue, RT order was CS (1,047) < AE (1,079) < GZ (1,084) < BJ (1,281). With encode duration cue, the ranking was GZ (1,039) < AE (1,076) < CS (1,084) < BJ (1,105). With encode intensity cue, the rank was CS (990) < GZ (1,075) < AE (1,078) < BJ (1,157). With process vowel reduction cue, the rank was BJ (1,024) < AE (1,158) < GZ (1,162) < CS (1,163). With all process cues, the rank was CS (974) < AE (1,045) < GZ (1,071) < BJ (1,132).

## Discussion

### L1 Transfer Effects on F0

Experimental results shed light on how Chinese learners encode English lexical stress. There were no significant differences between BJ, CS, and GZ participants and English participants when stress was signaled by F0 cue. This lack of difference suggests that the Chinese participants successfully transferred the use of F0 cues from the lexical tone in their L1 to learn or recognize English lexical stress.

Beijing, CS, and GZ participants did not differ from each other possibly due to the influence of L1 tone. F0 is generally considered the primary acoustic cue for distinguishing lexical tone (Fu et al., 1998). L1 features may help BJ, CS, and GZ participants perceive English lexical stress under the F0 condition. According to CWT, the more important an L1 cue is, the more it is used in L2 processing (Francis and Nusbaum, 2002; Holt and Lotto, 2004, 2006; Zhang and Francis, 2010; Qin et al., 2017).

F0 cue plays a robust role in lexical tones. If a language utilizes F0 for lexical distinction, it is unlikely for pitch to take on the extra-functional load for rhythmic distinction (Auer, 1993). Individual Chinese dialects should be more durationally than melodiously distinct because F0 is a constant in lexical tone. CS participants performed the best in F0 cue. It is possible that CS participants had sufficient experience using the F0 cue to signal stress (Yi, 2007). GZ learners perform well in F0 cue. This is probably because GZ dialect is a tone language with a relatively complicated tonal system. GZ participants were familiar with the varying F0 contour, which gave a great advantage in perceiving lexical stress in the F0 cue condition.

### L1 Transfer Effects on Duration

When stress was signaled only by duration cue, GZ participants performed less well in a sequence-encoding task than did BJ, CS, or AE participants. This suggests that when specific cues signal stress individually, L2 learners have different preferences when using these cues. They tended to use the cues that they were very familiar within their L1 dialect. For BJ and CS participants, duration cue was explored to encode a word stress distinction, such as to distinguish stressed and unstressed syllables. It was hypothesized that BJ and CS dialect tone duration preconditioned BJ and CS participants to encode English lexical stress in the duration condition.

Beijing, CS, and GZ participants performed worse than AE participants in the use of the duration cue. One possibility suggested that there is a greater likelihood of vowel reduction in English than in BJ, CS, or GZ dialect. The more frequent use of the duration cue with the tense and lax vowels, such as/i/and/I/, in English may result in AE participants being more sensitive to duration than Chinese participants in dealing with English lexical stress (Lin et al., 2013). BJ may have a lexical stress contrast (Duanmu, 2007). CS has fewer words with stress placement contrast than does AE (Yi, 2007). Duration in BJ and CS dialects but not GZ is utilized to process lexical stress. This feature may lead BJ and CS participants to rely less on the duration cue compared to AE participants. GZ dialect has no lexical stress, so GZ participants relied less on the duration cue than other cues compared to the AE participants.

### L1 Transfer Effects on Intensity

When stress was signaled only with intensity cue, no significant differences were found among groups. Participants were less accurate in the condition of intensity cue. These findings indicate that intensity is a constant cue. This is in accordance with Zhang and Francis (2010). Intensity was ranked the least in cuing English word stress (Beckman, 1986). In addition, intensity plays as the secondary cue for Mandarin lexical stress (Whalen and Xu, 1992). All the four language groups were less able to use the intensity cue probably resulting from the less important role that intensity cue plays in cuing both lexical tone in Chinese dialects and lexical stress in English (Zhang and Francis, 2010). A difference between the trochaic and iambic stress patterns was generally less clearly marked (Fry, 1955). Intensity cues varied widely from one speaker to another, not only in absolute values but also in the intensity ratio of the stressed to unstressed vowels.

### L1 Transfer Effects on Vowel Reduction

When stress was signaled by the vowel reduction cue, AE participants were more attentive to vowel reduction than other cues (such as F0, duration, and intensity). This is consistent with previous studies (Zhang and Francis, 2010). “In most cases, vowel reduction is more salient than other cues” (Zhang and Francis, 2010). Chinese participants used the vowel reduction cue to distinguish stress perhaps because they have used perception strategy regardless of their L1 tonal background. A specific prosodic cue, not necessarily a familiar cue to non-native speakers, which is utilized in their native category, may be applied by non-native speakers. The reason may be that this cue is easier to access than other prosodic cues in perception (Bohn, 1995). When stress is conveyed by other acoustic cues, such as intensity or duration, it is not sufficient for Chinese participants to differentiate English stress contrasts. The vowel reduction cue was utilized for distinguishing stress contrasts regardless of whether Chinese participants have prior experience with this cue.

Better BJ participant performance than CS and GZ dialect may be explained by the greater occurrence of vowel reduction in BJ dialect than in CS and GZ dialects. GZ participants are less able to use the vowel reduction cue in identifying English lexical stress identification task because of the less prominent role in the GZ dialect (Qin et al., 2017). One possibility is the greater occurrence of vowel reduction in AE than in Chinese dialects. Chinese dialect experience affects the participants’ perception of English vowel reduction. The frequency of the vowel/ǝ/occurs very often in English. Although both English and Chinese have vowel reduction generated by unstressed syllables, the frequency of vowel/ǝ/in Chinese is far less than that in English (Xu, 2008). The frequency of/ǝ/in the BJ dialect is relatively low. The frequency of/ǝ/is less in southern dialects. In the GZ dialect,/ǝ/almost no longer exists (Xu, 2008).

In addition, when processing English nonce words that contrasted in word initial/t/and/k/, the BJ, CS, and GZ dialect participants did not differ from each other, and all performed lower than the AE participants. The reason may be the differences in the phonetic realization of the target segments between English and Chinese dialects. For instance, English/t/is an alveolar, whereas Mandarin/t/is dental. Moreover, in their phonotactics, for example, lax vowels following/t/and/k/in the word do not occur in Chinese (Qin et al., 2017).

All groups were more accurate under the all-cue condition than under any single isolated cue condition. One possibility may be that the greater efforts of the stressed syllables under the all-cue condition than under any single cue condition. Under the all-cue condition, the stressed syllable is more salient than the unstressed syllable.

The results can be explained by CWT. The findings proved that the weighting of acoustic cues in L1 determines cue weight in L2 speech perception. This theory holds that if a specific acoustic cue plays an important role in distinguishing L1 lexical words, it can be used to encode L2 stress, even if an L1 has no lexical stress. On the contrary, if a specific acoustic cue plays a limited role in L1 lexical access, it should be difficult for L2 learners to use it in acquiring L2. This theory holds that the more important a cue is in L1, the more those learners will use it in L2 processing (Francis and Nusbaum, 2002; Holt and Lotto, 2004, 2006; Zhang and Francis, 2010; Qin et al., 2017). This study found that four groups performed similarly when processing nonce words in the F0 condition. BJ and CS participants were more accurate than GZ participants in duration cue and vowel reduction. These results are consistent with the prediction of CWT, that is, F0 is an important cue of Chinese lexical tones, which can be successfully used in L2 stress. As predicted by CWT, the more important cues are in the L1, the more they are used in L2 processing (Qin et al., 2017). CWT predicts under what circumstances L2 learners can or cannot process L2 lexical stress and how they process L2 English lexical stress to recognize L2 words.

Each acoustic cue of English lexical stress was controlled independently when preparing the conditions for the perception task conditions. This condition was different from those of prior studies (Qin et al., 2017), which focused on the two cues (F0 and duration) at a time. This study manipulated all four cues. This level of control was beneficial as it made more explicit how each variable affected the perceptions of intelligibility and nativeness. To have superior control, stimuli were manipulated and resynthesized. This is a successful attempt to use the manipulated words to investigate the weighting of the acoustic cues. This study conducted a perception experiment to make it possible to provide a more complete conclusion as to what exactly causes L1 dialect transfer to L2 learners’ perception of the acoustic cues.

## Conclusion

The experiment examined how AE participants and BJ, CS, and GZ participants performed in a sequence-encoding task. Those groups performed similarly when processing nonce words in the F0 condition. BJ and CS participants performed better than GZ participants in the duration and vowel reduction condition. BJ, CS, and GZ groups relied less on the duration cue condition than did AE participants. BJ, CS, and GZ participants more relied on the F0 cue due to the influence of lexical tone. RT suggests that the processing time of acoustic cues across different language groups was significantly different. Four groups displayed different speeds in identifying the English lexical stress, suggesting that they use different strategies in perception. These results were interpreted by CWT. The results suggest that the L1 dialect effect will transfer to the perception of English lexical stress with L2 learners.

One contribution of this study proves that it suggests that L1 dialect plays an important role in determining whether L2 learners can use specific acoustic cues to perceive English lexical stress. Many studies have focused on how L1 affects L2 stress acquisition (Zhang and Francis, 2010; Lin et al., 2013). Little research has explored how L1 dialects influence L2 English lexical stress. Another contribution is that it provides ideas and inspiration for improving teaching and learning efficiency of English lexical stress. Teachers could guide L2 learners to reassign acoustic cue weights and learn to build a target perceptual space. This study contributes to English L2 pedagogy and other fields such as speech disorders, word recognition, and pronunciation. The potential application of the results here could benefit L2 learners with dialect backgrounds or those with speech disorders as they strive to successfully perceive English. The results suggest that focusing primarily on vowel duration and intensity is most beneficial for language learners as these cues are mostly obviously related to perceived intelligibility and nativeness. The present study provides practical implications for teaching stress perception to Chinese learners of English from different dialect backgrounds, and possibly also for educating tone perception to AE learners of Chinese. Information of this nature may apply in clinical settings for improving the efficacy of language rehabilitation in bilingual speakers. Dialect participants should pay more attention to the duration cue.

This study also has some limitations. First, it covers only a small number of dialects. Second, only two-syllable structures confined to disyllabic nonce words were used. The resynthesized speech may not sound entirely natural, particularly due to F0 changes of the stimulus made the words sound more computerized. Third, only intermediate English proficiency level participants were used. Fourth, this study only explored the perception of English lexical stress.

This study uses scientific methods and techniques to explore the perception of English lexical stress to obtain more reliable insights. Many possibilities for future research related to the perception were encountered. First, more cross-dialect research is necessary to investigate whether dialect transfer exists in L2 study. Future research is needed to determine whether, and to what extent, this holds true among different learner populations and in diverse areas of L2 use. Including those from Chinese Ethnic Minorities to reveal the effects of L1 rhythm typologies on a more extensive scale. Second, various syllable structures and more syllables should be utilized to learn more about the processes of English lexical stress acquisition. Real English words unfamiliar to the learners can also be tested. English lexical stress existing in spontaneous discourses can be studied to yield a full picture reflecting the aspects of acoustic parameters in English lexical stress. Moreover, research on sentential stress would be a meaningful direction. Third, the effect of proficiency in L2 stress acquisition merits further investigation. Fourth, it is expected that the Chinese dialect participants may exhibit differences in the acoustic realization of English lexical stress. The relationship between lexical stress perception and production is worthy of further exploration.

In conclusion, the results reveal that L1 native dialect background is a potentially influential factor, which transfers in L2 speech perception. It is suggested that exploring the impact of L1 dialect on L2 stress acquisition is very important for L2 acquisition theories and L2 stress teaching. The fact that Chinese who were learners of English failed to realize English contrasts, does not mean that they cannot encode these necessary acoustic correlates, but that explicitly teaching of these cues is crucial during the preliminary stage of learning English stress. Explicit training of L2 learners of these acoustic correlates will promote learners to accurately master the cues more quickly than leaving it up to the learners to ascertain the characteristics through exposure to L2. It is necessary to construct L1–L2 relationships at different levels of analysis, to predict which L2 elements constitute difficulties for learners of different L1 dialect backgrounds.

## Data Availability Statement

The original contributions presented in the study are included in the article/Supplementary Material, further inquiries can be directed to the corresponding author/s.

## Ethics Statement

Ethical review and approval were not required for the study on human participants in accordance with the local legislation and institutional requirements. The participants provided their written informed consent to participate in this study.

## Author Contributions

XG proposed the research questions, designed the experiment, collected data, conducted statistical analysis, and wrote the draft of the manuscript. XC provided valuable suggestions and revised it. Both authors have approved the version of this manuscript.

## Conflict of Interest

The authors declare that the research was conducted in the absence of any commercial or financial relationships that could be construed as a potential conflict of interest.

## Publisher’s Note

All claims expressed in this article are solely those of the authors and do not necessarily represent those of their affiliated organizations, or those of the publisher, the editors and the reviewers. Any product that may be evaluated in this article, or claim that may be made by its manufacturer, is not guaranteed or endorsed by the publisher.
