# Supplementary material for: Perception of English Stress of Synthesized Words by Three Chinese Dialect Groups
Source: Front Psychol. 2022 Mar 16;13:803008. doi: 10.3389/fpsyg.2022.803008 (PMC8966649; doi:10.3389/fpsyg.2022.803008)
Supplement: Supplementary file 1 [file Data_Sheet_1.docx]

Supplementary Material

## Supplementary Tables

**Table** **1 Comparison of tones in Beijing, Changsha and Guangzhou dialects**

| **Language family** | **Dialect** | **Number of tones** | **Tones number** | **Tone name** | **5-level** | **Example (meaning)** |
| --- | --- | --- | --- | --- | --- | --- |
| Northern dialect | BJ dialect | 4 | T1 | High level | 55 | 妈ma55 (mother) |
|  |  |  | T2 | High rising | 35 | 麻ma35(hemp) |
|  |  |  | T3 | Low-Dipping | 214 | 马ma214(horse) |
|  |  |  | T4 | High falling | 51 | 骂ma51(scold) |
| Southern dialect | CS dialect | 6 | T1 | Mid-level | 33 | 巴/pa33/ (cling to) |
|  |  |  | T2 | Low rising | 13 | 爬/pa13/ (climb) |
|  |  |  | T3 | Falling tone | 31 | 把/pa31/ (control) |
|  |  |  | T4 | High level | 45 | 霸/pa45/ ([domination](javascript:void(0);)) |
|  |  |  | T5 | Low level | 21 | 稗/pa21/ (panicum crus-galli L) |
|  |  |  | T6 | Checked tone | 24 | 八/pa24/(eight） |
|  | GZ dialect^b^ | 9 | T1^a^ | High level | 55 | 诗/si1/(poet) |
|  |  |  | T2 | Mid rising | 25 | 史/si2/(history) |
|  |  |  | T3 | Mid-level | 33 | 试/si3/(test) |
|  |  |  | T4 | Low falling | 21 | 时/si4/(time) |
|  |  |  | T5 | Low rising | 13 | 市/si5/(city) |
|  |  |  | T6 | Low level | 22 | 事/si6/(matter) |
|  |  |  | T7 | High level | 5 | 色/si7/(colour) |
|  |  |  | T8 | Mid-level | 3 | 锡/si8/(tin) |
|  |  |  | T9 | Low level | 2 | 食/si9/(food) |

*Note.* T1-T4 of Changsha and Guangzhou dialects are different from those of Mandarin. Neutral tone is precluded in this table.

^a^ There are two different types of T1 spoken by Guangzhou speakers, namely: 55 (high level) and 53 (high falling). Young Guangzhou dialect speakers inclined to combine 53 into 55. This study group them together into T1.

^b^ T2, T4, and T5 of Guangzhou dialect can also be coded as 35, 11 and 23, respectively. Generally speaking, Cantonese is a tonal language with 6 phonetic tones (Alexander et al., 2008). T7, T8 and T9 were considered as “checked tones”, identifying Cantonese with 9 tones. They are relatively shorter in duration in the F0 contour.

**Table** **2 Self-evaluation of English proficiency of three Chinese dialect groups**

|  | **Degree of practice with English** | | | **Mean** |
| --- | --- | --- | --- | --- |
|  | **BJ** | **CS** | **GZ** |  |
| Pronunciation (1: Extremely poor- 10: Perfect) | | | | |
| Mean | 6.35 | 6.17 | 6.03 | 6.18 |
| SE | 0.42 | 0.44 | 0.56 | 0.47 |
| Vocabulary (1: Extremely poor- 10: Perfect) | | | | |
| Mean | 6.89 | 6.43 | 6.98 | 6.77 |
| SE | 0.37 | 0.54 | 0.46 | 0.46 |
| Grammar (1: Extremely poor- 10: Perfect) | | | | |
| Mean | 6.87 | 6.41 | 6.73 | 6.67 |
| SE | 0.44 | 0.34 | 0.54 | 0.44 |

**Table** **3 Participants’ demographics**

| **Group** | **Gender (F/M)** | **Age^a^**  **(s.d.)** | **AOA^b^**  **(s.d.)** | **DEU^c^**  **(s.d.)** | **DMU^d^**  **(s.d.)** | **DDU^e^**  **(s.d.)** | **Proficiency^f^**  **(s.d.)** | **LexTALE^g^**  **(s.d.)** |
| --- | --- | --- | --- | --- | --- | --- | --- | --- |
| BJ | 10/10 | 20.2  (2.2) | 13.1 (2.5) | 14.1 (3.0) | 38.5  (0.8) | 47.4  (0.3) | 6.7  (1.2) | 66.4  (3.4) |
| CS | 10/10 | 17.2  (2.0) | 10.4  (2.2) | 11.3 (7.5) | 32.5  (2.1) | 56.2  (0.5) | 6.1  (2.3) | 60.7  (2.5) |
| GZ | 10/10 | 18.8  (1.9) | 10.2 (2.3) | 9.3 (4.3) | 31.7  (0.7) | 59.0  (0.2) | 5.8  (1.6) | 57.9  (2.3) |
| AE | 10/10 | 22.7  (2.9) | -- | -- | -- | -- | -- | -- |

s.d.: standard deviation.

^a^Age: mean age when testing (in years).

^b^AOA: mean age of English acquisition (in years).

^c^DEU: daily English usage (in percentage).

^d^DMU: daily Mandarin usage (in percentage).

^e^DDU: daily dialect usage (in percentage.

^f^Proficiency: the proficiency evaluated by themselves (10-point Likert scale, 0 = “none” ; 10 = “perfect”).

^g^ LexTALE score was converted to a percentage.

**Table** **4 Experimental stimuli** (adapted from Wang (2016) and Qin et al. (2017))

| **Disyllabic Words** | **C_1_** | **V_1_** | **C_2_** | **V_2_** |
| --- | --- | --- | --- | --- |
| /'sɪvi/ | s | I  ʊ  ʌ | v | i |
| /'zʊθi/ | z |  | θ |  |
| /'fʌði/ | f |  | ð |  |
| /'hʌfi/ | h |  | f |  |
| /sɪ'vi/ | s | I  ʊ  ʌ | v |  |
| /zʊ'θi/ | z |  | θ |  |
| /fʌ'ði/ | f |  | ð |  |
| /hʌ'fi/ | h |  | f |  |
| /'savə/ | s | a | v | ə |
| /'zaθə/ | z |  | θ |  |
| /'fasə/ | f |  | ð |  |
| /'hafə/ | h |  | f |  |
| /sə'va/ | s | ə | v | a |
| /zə'θa/ | z |  | θ |  |
| /fə'sa/ | f |  | ð |  |
| /hə'fa/ | h |  | f |  |

**Table** **5 Filler stimuli** (adapted from Wang (2016) and Qin et al. (2017))

| **Disyllabic Words 1** | **Disyllabic Words 2** | **C_1_** | **V_1_** | **C_2_** | **V_2_** |
| --- | --- | --- | --- | --- | --- |
| /'tɪbi/ | /'kɪbi/ | t/k | I  ʊ  ʌ | b | i |
| /'tʊdi/ | /kʊdi/ | t/k |  | d |  |
| /'fʌgi/ | /'kʌgi/ | t/k |  | g |  |
| /'tʌmi/ | /'kʌmi/ | t/k |  | m |  |
| /tɪ'bi/ | /kɪ'bi/ | t/k | I  ʊ  ʌ | b | i |
| /tʊ'di/ | /kʊ'di/ | t/k |  | d |  |
| /tʌ'gi/ | /kʌ'gi/ | t/k |  | g |  |
| /tʌ'mi/ | /kʌ'mi/ | t/k |  | m |  |
| /'tafə/ | /'kafə/ | t/k | a | f | ə |
| /'tadə/ | /kavə/ | t/k |  | v |  |
| /'tagə/ | /'kaθə/ | t/k |  | θ |  |
| /'tamə/ | /'ka ðə/ | t/k |  | ð |  |
| /tə'ba/ | /kə'fa/ | t/k | ə | f | a |
| /tə'da/ | /kə'va/ | t/k |  | v |  |
| /tə'da/ | /kə'θa/ | t/k |  | θ |  |
| /tə'da/ | /kə' ða/ | t/k |  | ð |  |

**Table** **6 Logit mixed-effects models on all speakers’ accuracy on filler stimuli**

| **Variable** | **Est.** | ***SE*** | ***z*** | ***p*** |
| --- | --- | --- | --- | --- |
| Intercept | 2.1 | 0.38 | 5.2 | < 0.0001 |
| L1 (BJ) | -1.4 | 0.47 | -2.5 | < 0.001 |
| L1 (CS) | -1.0 | 0.47 | -2.5 | < 0.001 |
| L1 (GZ) | -0.9 | 0.46 | -2.5 | < 0.001 |

Note: The English group’s performance was the baseline.

L1: first language.

**Table** **7 Accuracy in the sequence recall task**

| **Cue * Group** | | | | | |
| --- | --- | --- | --- | --- | --- |
| Dependent Variable: Accuracy | | | | | |
| Cue | Group | Mean | Std. Error | 95% Confidence Interval | |
|  |  |  |  | Lower Bound | Upper Bound |
| F0 | AE | .583 | .044 | .498 | .669 |
|  | BJ | .500 | .013 | .244 | .756 |
|  | CS | .635 | .046 | .545 | .726 |
|  | GZ | .525 | .032 | .462 | .587 |
| Duration | AE | .398 | .022 | .313 | .484 |
|  | BJ | .280 | .031 | -.24 | .506 |
|  | CS | .240 | .026 | .149 | .330 |
|  | GZ | .219 | .072 | .206 | .381 |
| Intensity | AE | .231 | .032 | .146 | .317 |
|  | BJ | .250 | .021 | .116 | .506 |
|  | CS | .229 | .023 | .139 | .320 |
|  | GZ | .196 | .027 | .134 | .258 |
| Vowel reduction | AE | .544 | .080 | .359 | .590 |
|  | BJ | .483 | .041 | .373 | .340 |
|  | CS | .490 | .066 | .399 | .580 |
|  | GZ | .417 | .082 | .354 | .479 |
| All | AE | .767 | .065 | .581 | .792 |
|  | BJ | .750 | .423 | .494 | 1.006 |
|  | CS | .542 | .033 | .451 | .632 |
|  | GZ | .623 | .044 | .560 | .685 |

**Table** **8 Logit mixed-effects models on all participants’ accuracy on the experimental stimuli**

| **Variable** | **Est.** | **SE** | ***z*** | ***p*** |
| --- | --- | --- | --- | --- |
| Intercept | -2.06 | 0.45 | -4.62 | < 0.001*** |
| Cue (F0) | 2.46 | 0.37 | 6.73 | < 0.001*** |
| Cue (Duration) | 1.48 | 0.36 | 4.13 | < 0.001*** |
| Cue (Intensity) | 0.52 | 0.37 | 1.38 | 0.17 |
| Cue (Vowel reduction) | 1.75 | 0.36 | 4.88 | < 0.001*** |
| Cue (all cues) | 2.93 | 0.38 | 7.79 | < 0.001*** |
| L1 (BJ) | -13.51 | 677.88 | -0.02 | 0.98 |
| L1 (CS) | 0.50 | 0.59 | 0.86 | 0.39 |
| L1 (GZ) | -0.28 | 0.53 | -0.54 | 0.59 |
| Cue (F0) ×L1 (BJ) | 13.11 | 677.88 | 0.019 | 0.98 |
| Cue (Duration) ×L1 (BJ) | 12.93 | 677.88 | 0.019 | 0.98 |
| Cue (Intensity) ×L1 (BJ) | 13.89 | 677.88 | 0.020 | 0.98 |
| Cue (Vowel reduction) ×L1 (BJ) | 11.35 | 677.88 | 0.017 | 0.99 |
| Cue (all cues) ×L1 (BJ) | 13.80 | 677.88 | 0.020 | 0.98 |
| Cue (F0) ×L1 (CS) | -0.25 | 0.51 | -0.50 | 0.62 |
| Cue (Duration) ×L1 (CS) | -1.23 | 0.52 | -2.39 | 0.02* |
| Cue (Intensity) ×L1 (CS) | -0.33 | 0.52 | -0.63 | 0.53 |
| Cue (Vowel reduction) ×L1 (CS) | -0.20 | 0.50 | -0.40 | 0.69 |
| Cue (all cues) ×L1 (CS) | -1.16 | 0.51 | -2.27 | 0.02* |
| Cue (F0) ×L1 (GZ) | -0.003 | 0.46 | -0.006 | 0.995 |
| Cue (Duration) ×L1 (GZ) | -0.039 | 0.45 | -0.09 | 0.93 |
| Cue (Intensity) ×L1 (GZ) | 0.17 | 0.48 | 0.37 | 0.72 |
| Cue (Vowel reduction) ×L1 (GZ) | 0.21 | 0.45 | 0.47 | 0.64 |
| Cue (all cues) ×L1 (GZ) | 0.01 | 0.47 | 0.02 | 0.98 |

Note. Significance codes: “***”*p ≦* 0.001; “**”*p* ≦ 0.01; ‘*’ *p* ≦ 0.05; ‘.’*p* ≦ 0.1“ ”*p* > 1.0.

**Table** **9 Logit mixed-effects models on the L2 learners’ sequence-encoding accuracy on the experimental stimuli**

| **Variable** | **Est.** | **SE** | ***z*** | ***p*** |
| --- | --- | --- | --- | --- |
| Intercept | -2.03 | 0.40 | -5.03 | < 0.0001*** |
| Cue (F0) | 2.41 | 0.36 | 6.70 | < 0.0001*** |
| Cue (Duration) | 1.47 | 0.35 | 4.15 | < 0.0001*** |
| Cue (Intensity) | 0.53 | 0.37 | 1.44 | 0.15 |
| Cue (Vowel reduction) | 1.70 | 0.35 | 4.82 | < 0.0001*** |
| Cue (all cues) | 2.87 | 0.37 | 7.75 | < 0.0001*** |
| L1 (BJ) | -13.55 | 695.33 | -0.02 | 0.98 |
| L1 (CS) | 0.39 | 0.57 | 0.86 | 0.39 |
| L1 (GZ) | -0.28 | 0.51 | -0.54 | 0.59 |
| Cue (F0) ×L1 (BJ) | 13.16 | 695.33 | 0.019 | 0.98 |
| Cue (Duration) ×L1 (BJ) | 12.99 | 695.33 | 0.019 | 0.99 |
| Cue (Intensity) ×L1 (BJ) | 13.92 | 695.33 | 0.020 | 0.98 |
| Cue (Vowel reduction) ×L1 (BJ) | 11.44 | 695.33 | 0.016 | 0.99 |
| Cue (all cues) ×L1 (BJ) | -1.13 | 0.51 | -2.23 | 0.03* |
| Cue (F0) ×L1 (CS) | -0.25 | 0.50 | -0.49 | 0.63 |
| Cue (Duration) ×L1 (CS) | -1.20 | 0.51 | -2.35 | 0.02* |
| Cue (Intensity) ×L1 (CS) | -0.32 | 0.52 | -0.62 | 0.54 |
| Cue (Vowel reduction) ×L1 (CS) | -0.19 | 0.49 | -0.39 | 0.69 |
| Cue (all cues) ×L1 (CS) | -1.13 | 0.51 | -2.23 | 0.03* |
| Cue (F0) ×L1 (GZ) | 0.001 | 0.45 | 0.003 | 0.99 |
| Cue (Duration) ×L1 (GZ) | -0.039 | 0.45 | -0.09 | 0.93 |
| Cue (Intensity) ×L1 (GZ) | 0.17 | 0.47 | 0.36 | 0.72 |
| Cue (Vowel reduction) ×L1 (GZ) | 0.21 | 0.45 | 0.46 | 0.64 |
| Cue (all cues) ×L1 (GZ) | 0.01 | 0.46 | 0.02 | 0.98 |

Note. Significance codes: “***”*p ≦* 0.001; “**”*p* ≦ 0.01; ‘*’ *p* ≦ 0.05; ‘.’*p* ≦ 0.1“ ”*p* > 1.0.

**Table** **10 RT in the five conditions in the perception experiment**

| **Cue * Group** | | | | | |
| --- | --- | --- | --- | --- | --- |
| Dependent Variable: RT | | | | | |
| **Cue** | **Group** | **Mean** | **Std. Error** | **95% Confidence Interval** | |
|  |  |  |  | **Lower Bound** | **Upper Bound** |
| F0 | AE | 1079.185 | 37.321 | 1006.002 | 1152.368 |
|  | BJ | 1281.167 | 111.962 | 1061.618 | 1500.715 |
|  | CS | 1046.542 | 39.585 | 968.920 | 1124.164 |
|  | GZ | 1083.701 | 27.155 | 1030.453 | 1136.949 |
| Duration | AE | 1076.435 | 37.321 | 1003.252 | 1149.618 |
|  | BJ | 1105.083 | 111.962 | 885.535 | 1324.632 |
|  | CS | 1083.823 | 39.585 | 1006.201 | 1161.445 |
|  | GZ | 1038.554 | 27.155 | 985.306 | 1091.802 |
| Intensity | AE | 1077.852 | 37.321 | 1004.669 | 1151.035 |
|  | BJ | 1157.083 | 111.962 | 937.535 | 1376.632 |
|  | CS | 989.771 | 39.585 | 912.149 | 1067.393 |
|  | GZ | 1074.887 | 27.155 | 1021.639 | 1128.136 |
| Vowel reduction | AE | 1158.250 | 37.321 | 1085.067 | 1231.433 |
|  | BJ | 1023.917 | 111.962 | 804.368 | 1243.465 |
|  | CS | 1163.833 | 39.585 | 1086.211 | 1241.455 |
|  | GZ | 1162.069 | 27.155 | 1108.820 | 1215.317 |
| All | AE | 1044.509 | 37.321 | 971.326 | 1117.692 |
|  | BJ | 1132.083 | 111.962 | 912.535 | 1351.632 |
|  | CS | 973.969 | 39.585 | 896.347 | 1051.591 |
|  | GZ | 1071.480 | 27.155 | 1018.232 | 1124.729 |
